# In-depth cellular and humoral dynamics of the response to COVID-19 vaccine booster in patients with chronic B-cell neoplasms

**DOI:** 10.1038/s41408-023-00884-w

**Published:** 2023-07-26

**Authors:** Emily Ayers, Glenda Canderan, Michael E. Williams, Behnam Keshavarz, Craig A. Portell, Jeffrey M. Wilson, Judith A. Woodfolk

**Affiliations:** 1 Division of Hematology and Oncology and Comprehensive Cancer Center, University of Virginia School of Medicine, Charlottesville, VA USA; 2 Division of Asthma, Allergy and Immunology, Department of Medicine, University of Virginia School of Medicine, Charlottesville, VA USA

**Keywords:** Immunological memory, B-cell lymphoma

Dear Editor,

While the development of SARS-CoV-2 vaccines offers substantial protection against COVID-19 illness in the general population, impaired antibody responses are present in patients with mature B-cell neoplasms, including chronic lymphocytic leukemia (CLL) [1]. This is the case regardless of treatment status, although B-cell targeting agents, such as anti-CD20 mAb and BTK inhibitor therapy, further inhibit antibody responses [2, 3]. Despite this, antibody responses may improve with repeated vaccinations [4, 5]. By contrast, data regarding T-cell responses in these patients is less clear. Vaccine-induced memory T cells are essential for providing help to B cells for antibody production, as well as aiding in viral clearance upon subsequent exposure. Several studies of vaccinated patients with hematologic malignancies have reported the presence of virus-specific T cells, even in the absence of a humoral response [6–8]. Such T cells harbor or secrete IFN-γ and, similar to antibodies, T-cell responses may be boosted with repeated vaccinations [4, 5, 8, 9].

Little is known about the cellular features that differentiate a successful antibody response from one that is deficient in patients with lymphoid malignancies. Here, we used high-dimensional single-cell profiling coupled with machine learning to define the cellular landscape before and after SARS-CoV-2 mRNA vaccine booster (dose 3) in patients with CLL.

As a first step, antibody responses were analyzed between 12 and 50 days post-vaccine booster in 56 patients with non-Hodgkin lymphoma (NHL), most of whom received a homologous vaccination series with BNT162b2 (*n* = 26, 46%) or mRNA-1273 (*n* = 25, 45%) (Table S1). Hematologic diagnoses included CLL (45%), mantle cell lymphoma (MCL, 20%), diffuse large B-cell lymphoma (DLBCL, 9%), follicular lymphoma (FL, 9%), marginal zone lymphoma (MZL, 9%) and Waldenstrom macroglobulinemia (WM, 9%). Treatments included anti-CD20 monoclonal antibodies in 9 (16%), BTK inhibitors in 11 (20%), and other treatments in 12 (21%). Twenty-five patients (45%) were not currently receiving therapy, and most were treatment-naive. NHL patients were similar in age and received similar frequencies of COVID-19 vaccines as compared to a healthy reference cohort (Table S2). IgG antibody responses to SARS-CoV-2 spike RBD (S-RBD) were lower in NHL patients versus healthy controls at all time points (pre-booster, and at ~3 weeks and ~6 months post-booster) (Fig. 1A). Moreover, in contrast with healthy controls, antibody levels did not significantly increase after vaccine booster in NHL patients (*p* = 0.16 versus *p* < 0.001). Almost half of patients (48%) were considered antibody responders on the basis of post-booster IgG levels to S-RBD > 1 µg/mL and were more likely than non-responders to be treatment-naive (44% vs 14%, *p* = 0.02) (Table S1). Among patients on anti-CD20 and BTKi therapy, responder rates were 33% and 36%, respectively. Interestingly, whereas all subjects with MZL responded, none with FL responded (Fig. 1B), despite similar treatment types and median time from last treatment dose (Table S1 and data not shown). Levels of anti-S-RBD IgG at ~3 weeks and ~6 months post-booster were markedly higher in patients who had higher antibody levels (>1 µg/mL) prior to booster versus those with lower levels (Fig. 1C).

**Fig. 1:**
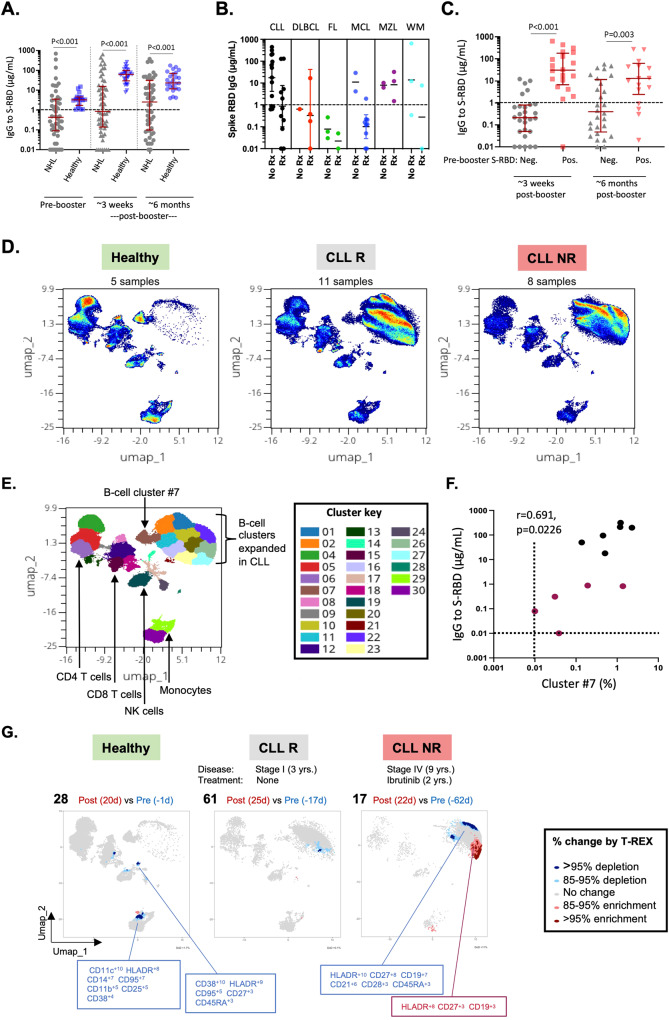
Antibody responses to SARS-CoV-2 vaccine booster and the B-cell landscape in patients with chronic B-cell neoplasms. **A** Levels of serum IgG to S-RBD in NHL patients (*n* = 56) and a healthy cohort (*n* = 28) measured by ImmunoCAP assay [15]. **B** Levels of IgG stratified by NHL diagnosis and treatment status. Numbers for treated (Rx) and non-treated (No Rx) subjects, respectively, were as follows: CLL: 11 and 14; DLBCL: 4 and 1; FL: 2 and 3; MCL: 9 and 2; MZL: 3 and 2; WM: 2 and 3. Error bars are shown only for more than 3 data points. No Rx patients includes patients not on therapy at time of vaccination. **C** Levels of IgG to S-RBD in NHL patients stratified by response prior to vaccine booster. Numbers for negative and positive subgroups were 28 and 20, respectively. Negative (Neg.) subgroups included subjects who had <1 μg/ml IgG to S-RBD at the pre-booster time point. **D** Visualization of high-dimensional flow cytometry data by Uniform Manifold Approximation and Projection (UMAP). Data is shown for total leukocytes in pooled samples (pre- and post-vaccine booster) from healthy subjects (*n* = 3), CLL responders (CLL R, *n* = 6), and CLL non-responders (CLL NR, *n* = 6) analyzed by spectral flow cytometry using a 31-marker panel. Samples from subjects #50 and #159 (pre), and #50, #59, #60, and #64 (post) were excluded owing to low cell viability. **E** FlowSOM analysis of cell clusters for pooled samples from all 3 subject groups. Cell types within major islands are annotated to aid in interpretation. Discrete molecular signatures with corresponding number labels are denoted by colors within the UMAP and cluster key (see also Fig. S2). **F** Correlation between levels of IgG to S-RBD post-booster and percentages of cells in B-cell cluster #7 existing before booster. Black and magenta symbols denote responders and non-responder patients with CLL, respectively. Zero values were set to 0.01. **G** Analysis of cell dynamics over time using the T-REX algorithm. Representative data is shown for 3 subjects. Values in parentheses related to disease and treatment are for duration of disease and time on current treatment, respectively. Enrichment (red shading) and depletion (blue shading) of discrete cell clusters is depicted according to the percentage change for each cluster. Signatures of cell clusters were assigned by marker enrichment modeling, which scores each marker on a scale of 1–10 based on its enrichment within each cluster.

Next, we assessed cellular responses in relation to vaccine-induced antibodies in a subset of 12 patients who had CLL, and who were selected based on sufficient sample size for high-dimensional single-cell analyses to compare responders and non-responders to vaccine booster (*n* = 6 per group) (Table S3). Among non-responders, 3 were on BTKi treatment (2 Ibrutinib, 1 acalabrutinib) and 3 were treatment-naive, whereas all responders were treatment-naive. To assess the global immune landscape, a 31-marker panel (Table S4) was used to analyze the signatures of major cell types in the blood by spectral flow cytometry. As expected, initial inspection of total lymphocytes by manual gating of flow cytometry data revealed higher percentages of B cells in patients with CLL versus healthy controls (Fig. S1). High-dimensional analysis of compiled single-cell data corresponding to pre- and post-booster specimens revealed a marked decrease in a discrete population of naive B cells in CLL (cluster #7 – HLA-DR^+^IgD^+^CD19^+^CD21^+^CD45RA^+^CD1c^lo^CD38^lo^, *p* = 0.021), and its profound loss in non-responders (Figs. 1D, E, S2–4). Notably, the percentage of cells in cluster #7 existing pre-booster correlated with IgG antibodies to S-RBD post-booster (Fig. 1F). This finding echoed a previous report wherein numbers of CD19^+^IgD^+^CD27^-^ naive B cells correlated with vaccine-induced antibodies in immunocompromised subjects [10]. Moreover, in patients with CLL, multiple B-cell clusters, most of which co-expressed IgD and CD27, and were defined by differential expression of CD21, CD27, CD25, IgD, CD45RA, and CD11c, were markedly expanded as compared with healthy subjects (Fig. 1D, E, S3). Although lack of CD5 in our marker panel precluded labeling these clusters as neoplastic, their relative absence in healthy subjects and different distributions across CLL patients (Fig. S3C) strongly suggest it. These perturbations were accompanied by decreases in percentages of a prominent CD4^+^ transitional memory subset (cluster #4: CD45RA^lo^CD27^+^CCR7^lo^CD28^+^, *p* = 0.009) and naive CD8^+^ T cells (cluster #8: CD45RA^+^CCR7^+^CD27^+^, *p* = 0.019), as well as other immune cell types (clusters #28, #29, & #30, *p* ≤ 0.03) (Figs. 1D, E, S2 and S4).

Use of the T-REX algorithm [11] to analyze B-cell dynamics over time within individual patients with CLL revealed that B-cell cluster #7 remained unchanged in responders, and was consistently lacking in non-responder patients on BTKi therapy (Fig. 1G, Fig. S5). By contrast, expansion of discrete naive (IgD^+^) and memory (CD27^+^) B-cell clusters was a prominent feature of 3 non-responders on BTKi therapy, indicating ongoing perturbations in the B-cell compartment (Fig. 1G, Fig. S5).

Next, to assess whether virus-specific T cells were induced by mRNA vaccine in CLL patients, T cells responding to pooled peptides of SARS-CoV-2 spike protein (S) and nucleoprotein were analyzed by Activation Induced Marker (AIM) assay [12]. S-specific CD4^+^ T cells were detected in all CLL samples but one (subject #17, pre-booster sample), and at frequencies ranging from 0.08% to 4.05% of total CD4^+^ T cells (Figs. 2A and S6). Notably, there was no difference between antibody responder and non-responder groups, and frequencies in CLL patients were similar or else higher as compared to healthy controls. After vaccine booster, frequencies of S-specific T cells were generally increased, including in 2 patients who were non-responders receiving BTKi treatment. By contrast, nucleoprotein-specific CD4^+^ T cells were detected in only a single subject and at one time point, indicating that subjects were likely never infected with SARS-CoV-2 and that S-specific T cells detected were vaccine-induced. Similar findings were observed for S-specific CD8^+^ T cells (Figs. 2A and S6). Subsets of both S-specific CD4^+^ and CD8^+^ T cells expressed the lung-homing chemokine receptor CCR5 (Fig. S7). Moreover, the memory signatures of these cells were similar for patients with CLL and controls, and akin to those targeting common pathogens (see CEFX response in Fig. S7B); however, their phenotype was distinct from PHA-stimulated T cells. No relationships were identified between percentages of virus-specific T cells after vaccine booster and the timing of sample collections or antibody levels (data not shown). Together, these findings demonstrated successful induction of T cells after vaccine booster despite ongoing B-cell perturbations in CLL, and their similar features to those likely induced prior to illness against other microbial antigens.

**Fig. 2:**
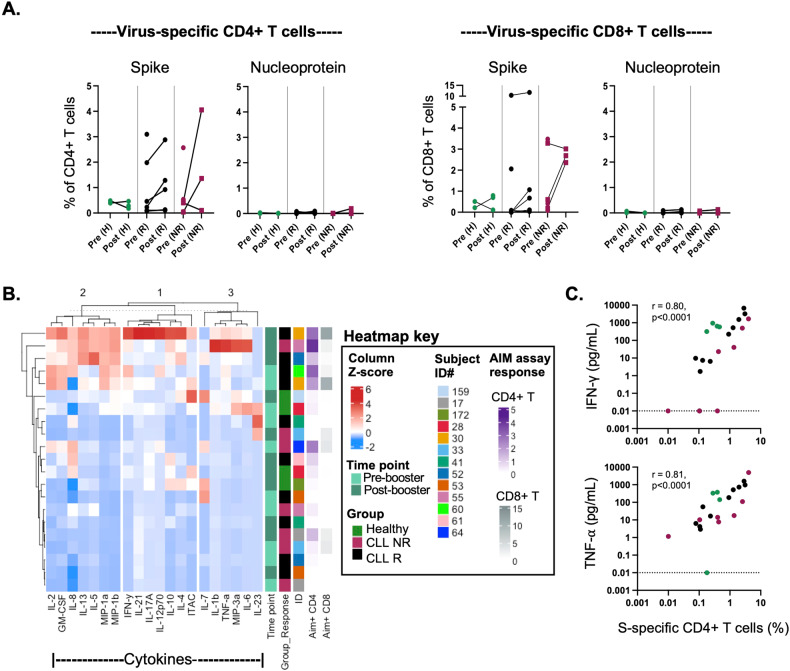
Functional virus-specific CD4^+^ and CD8^+^ T cells are present in CCL subjects after vaccine booster. **A** Frequencies of SARS-CoV-2-specific CD4^+^ T cells (OX40^+^CD137^+^) and CD8^+^ T cells (CD69^+^CD137^+^) detected by AIM assay after 24 h. in vitro stimulation with pooled peptides of spike protein and nucleoprotein. Percentages were derived by subtracting background values for unstimulated cultures. Data is shown for healthy subjects (H, green), CLL responders (R, black), and CLL non-responders (NR, magenta). Treated (BTKi) and untreated subjects are denoted by squares and circles, respectively. T cells were analyzed for available time points before and after vaccine booster. **B** Heatmap showing z-scaled cytokine levels (pg/ml) in AIM assay supernatants harvested after stimulation with pooled peptides of spike protein. Each row corresponds to data for one sample. **C** Correlation between percentages of CD4^+^ Spike-specific T cells detected by AIM assay and cytokine levels in assay supernatants. AIM assay data was obtained by subtracting values for unstimulated cultures from antigen-stimulated cultures. Data is shown for pre- and post-booster samples from CLL responders (black), CLL non-responders (magenta), and healthy subjects (green). Samples from subjects #50 and #159 (pre), and #50, #59, #60, and #64 (post) were excluded owing to low cell viability. Data for 3 pre-booster samples (#172, #59, and #61) were excluded from analysis of cytokine data for technical reasons.

Cytokine profiles in AIM assay supernatants were highly variable, regardless of disease or vaccine responder status, with highest mediator levels detected post-booster in 2 responder CLL patients (#30 and #52) and one non-responder CLL patient (#55), supporting T-cell function (Fig. 2B). However, the lowest levels of mediators were also produced in CLL patients, regardless of vaccine response, although PHA responses indicated that T cells remained functional (Fig. S8). Analysis of individual mediators produced in response to stimulation with peptides of spike protein revealed no significant differences across groups, after adjusting for multiple comparisons; however, an overall time-effect was observed for increases in the T-cell chemoattractant ITAC/CXCL11 (*p* = 0.043; data not shown). Finally, frequencies of S-specific CD4^+^ T cells, correlated with levels of multiple cytokines with the strongest relationships identified for TNF-α and IFN-γ (*r* ≥ 0.80, *p* < 0.0001) (Fig. 2C). By contrast, frequencies of S-specific CD8^+^ T cells correlated only with TNF-α, IL-12p70, and MIP-1β (*r* ≥ 0.49, *p* < 0.05). Together, these results confirm vaccine-induced virus-specific CD4^+^ and CD8^+^ T cells in CLL, regardless of antibody production, and their link to anti-viral type 1 responses.

Limitations of our study included the lack of assessment of neutralizing activity of antibodies, and antibodies to SARS-CoV-2 nucleoprotein. Additionally, T-cell responses were not compared at the same time points after vaccine booster owing to variable sampling across patients. Nonetheless, time windows generally exceeded those for peak effector T-cell responses, and frequencies of virus-specific T cells are reported to be stable for several months after vaccination [13, 14]. It was also not possible to calculate absolute numbers of virus-specific T cells, since blood counts were not clinically indicated at the time of sample collection.

In summary, our findings provide new insight into the nature of humoral and T-cell responses to SARS-CoV-2 vaccine booster and in vivo cellular dynamics in patients who have chronic B-cell neoplasms. The results support the usefulness of vaccination in patients with CLL to boost anti-viral T cells, even in the absence of antibody responses, and shed new light on the determinants and variability of vaccine response. The differences in vaccine response between disease types warrant further investigation of the biology of adaptive responses in patients with distinct B-cell malignancies.

## Supplementary information

Supplementary Material and Methods

Supplementary Figures and tables

## References

[1] Morawska M (2022). Reasons and consequences of COVID-19 vaccine failure in patients with chronic lymphocytic leukemia. Eur J Haematol.

[2] Apostolidis SA, Kakara M, Painter MM, Goel RR, Mathew D, Lenzi K (2021). Cellular and humoral immune responses following SARS-CoV-2 mRNA vaccination in patients with multiple sclerosis on anti-CD20 therapy. Nat Med.

[3] Roeker LE, Knorr DA, Thompson MC, Nivar M, Lebowitz S, Peters N (2021). COVID-19 vaccine efficacy in patients with chronic lymphocytic leukemia. Leukemia.

[4] Benjamini O, Gershon R, Bar-Haim E, Lustig Y, Cohen H, Doolman R (2023). Cellular and humoral response to the fourth BNT162b2 mRNA COVID-19 vaccine dose in patients with CLL. Eur J Haematol.

[5] Shen Y, Freeman JA, Holland J, Naidu K, Solterbeck A, Van Bilsen N (2022). Multiple COVID-19 vaccine doses in CLL and MBL improve immune responses with progressive and high seroconversion. Blood.

[6] Liebers N, Speer C, Benning L, Bruch PM, Kraemer I, Meissner J (2022). Humoral and cellular responses after COVID-19 vaccination in anti-CD20-treated lymphoma patients. Blood.

[7] Jimenez M, Roldan E, Fernandez-Naval C, Villacampa G, Martinez-Gallo M, Medina-Gil D (2022). Cellular and humoral immunogenicity of the mRNA-1273 SARS-CoV-2 vaccine in patients with hematologic malignancies. Blood Adv.

[8] Re D, Seitz-Polski B, Brglez V, Carles M, Graca D, Benzaken S (2022). Humoral and cellular responses after a third dose of SARS-CoV-2 BNT162b2 vaccine in patients with lymphoid malignancies. Nat Commun.

[9] Haydu JE, Maron JS, Redd RA, Gallagher KME, Fischinger S, Barnes JA (2022). Humoral and cellular immunogenicity of SARS-CoV-2 vaccines in chronic lymphocytic leukemia: a prospective cohort study. Blood Adv.

[10] Schulz E, Hodl I, Forstner P, Hatzl S, Sareban N, Moritz M (2021). CD19+IgD+CD27- naive B cells as predictors of humoral response to COVID 19 mRNA vaccination in immunocompromised patients. Front Immunol.

[11] Barone SM, Paul AG, Muehling LM, Lannigan JA, Kwok WW, Turner RB (2021). Unsupervised machine learning reveals key immune cell subsets in COVID-19, rhinovirus infection, and cancer therapy. Elife.

[12] Grifoni A, Weiskopf D, Ramirez SI, Mateus J, Dan JM, Moderbacher CR (2020). Targets of T cell responses to SARS-CoV-2 coronavirus in humans with COVID-19 disease and unexposed individuals. Cell.

[13] Maringer Y, Nelde A, Schroeder SM, Schuhmacher J, Horber S, Peter A (2022). Durable spike-specific T cell responses after different COVID-19 vaccination regimens are not further enhanced by booster vaccination. Sci Immunol.

[14] Keshavarz B, Richards NE, Workman LJ, Patel J, Muehling LM, Canderan G (2022). Trajectory of IgG to SARS-CoV-2 after vaccination with BNT162b2 or mRNA-1273 in an employee cohort and comparison with natural infection. Front Immunol.

[15] Keshavarz B, Wiencek JR, Workman LJ, Straesser MD, Muehling LM, Canderan G (2021). Quantitative measurement of IgG to severe acute respiratory syndrome coronavirus-2 proteins using immunoCAP. Int Arch Allergy Immunol.

