# Supplementary material for: In-depth cellular and humoral dynamics of the response to COVID-19 vaccine booster in patients with chronic B-cell neoplasms
Source: Blood Cancer J. 2023 Jul 26;13(1):114. doi: 10.1038/s41408-023-00884-w (PMC10372059; doi:10.1038/s41408-023-00884-w)
Supplement: Supplementary file 1 — Supplementary Material and Methods [file 41408_2023_884_MOESM1_ESM.docx]

**In-Depth Cellular and Humoral Dynamics of the Response to COVID-19 Vaccine Booster in Patients with Chronic B-Cell Neoplasms.**

Emily Ayers, MD ^1^*, Glenda Canderan, PhD ^2^*, Michael E Williams, MD ^1^, Behnam Keshavarz, PhD ^2^, Craig A Portell ^1#^, MD, Jeffrey M Wilson, MD PhD ^2#^, Judith A Woodfolk, MBChB, PhD ^2#^

^1^Division of Hematology and Oncology and Comprehensive Cancer Center, and ^2^Division of Asthma, Allergy and Immunology, Department of Medicine, University of Virginia School of Medicine; Charlottesville, VA, USA. Corresponding authors e-mail addresses: eca2t@virginia.edu and gc8jw@virginia.edu

**Methods**

***Study Design***

This was a prospective, longitudinal study of patients with mature B-cell neoplasms receiving the commercially-available SARS-CoV-2 vaccines. Subjects were recruited from the clinics at the University of Virginia from March 2021 to December 2021. Subjects were eligible if they had a diagnosis of a mature B-cell neoplasm and were planning to receive a vaccine targeting SARS-CoV-2. Clinical information was obtained through review of electronic health records for enrolled subjects (**Table S1 and S2**). Analyses for this report were restricted to 56 subjects who had a sample available at 12-50 days after booster immunization following a primary vaccination series (two doses of BNT162b2, two doses of mRNA-1273 or a single dose of Ad26.CoV.S). Booster vaccine was received 6-7 months post-dose 2 of mRNA vaccines. Most patients had samples available within the 3 weeks preceding booster immunization. A reference cohort was comprised of 28 age-similar employees at the University of Virginia who participated in a COVID-19 vaccine antibody study, as previously reported (see **Table S2** for demographics) (1, 2). For the current analysis, inclusion of the reference cohort was restricted to all volunteers over 50 years of age who had a sample collected between 12-50 days after the booster immunization. All studies were approved by the University of Virginia Health Science Research Institutional Review Board with written informed consent of all study participants (protocols: UVA HSR200171 and HSR210070).

***Antibody Assays***

IgG antibodies to SARS-CoV-2 spike RBD (S-RBD) were evaluated at three time-intervals in relation to the booster vaccine: pre-booster (0 to 30 days prior to booster), ~ 3 weeks post-booster (days 12-50 post-booster) and ~ 6 months post-booster (days 120-240). Antibodies were measured with a high-capacity quantitative ImmunoCAP-based assay using a Phadia 250 (Thermo-Fisher/Phadia, Waltham, MA, USA), as previously described (3). In brief, commercially acquired recombinant coronavirus proteins were biotinylated and conjugated to the streptavidin-coated solid phase of the ImmunoCAP. Here the cut-off of the assay was considered as 1 µg/mL and values that were less than 0.01 µg/mL were assigned a value of 0.01 µg/mL for data analysis. Subjects were classified as antibody responders if their IgG level at 3 weeks post-booster was >1 µg/mL.

***Analysis of Immune Cells in the Blood***

Twelve subjects with CLL (ages 52 to 88 yrs) and 3 healthy controls were selected for cellular studies (**Table S3**). All lacked a clinical history of previous SARS-CoV-2 infection. PBMCs were isolated by density gradient centrifugation before dose 2 of mRNA vaccine (0 to 62 days before) and after dose 3 (12-40 days after). Cells were cryopreserved for later analysis.

***Deep Phenotyping of Immune Cells:*** PBMCs were analyzed using a 31-color antibody panel for spectral flow cytometry (**Table S4**) designed to capture innate and adaptive immune cell types in the blood. One million cells were stained in a 100 μl mix of monoclonal antibodies, Fc receptor block (Human TruStain FcX, Biolegend, San Diego, CA, USA), Brilliant violet buffer (BD Bioscience, San Diego, CA, USA ) and PBS containing 2% FBS. Cells were then fixed (Cytofix buffer (BD Bioscience, San Diego, CA, USA) and acquired by spectral flow cytometry (5-laser Cytek Aurora, Cytek, Fremont, CA, USA). Data was analyzed after unmixing/compensating data (compensation beads, BD Biosciences) and excluding dead cells (LIVE/Dead Fixable blue - stain kit, Invitrogen, Carlsbad, CA, USA).

***Detection of SARS-CoV-2-Specific T Cells:*** T cells responding to SARS-CoV-2 proteins were identified by activation induced marker (AIM) assay (4). Briefly, PBMCs were stimulated in RPMI (Gibco, Waltham, MA, USA) containing 5% human serum (Sigma, Darmstadt, Germany) for 24 hours (1 million cells per well in 96-well plates) using pooled 15-mer peptides spanning the entire length of spike glycoprotein and nucleoprotein (1 μg/ml, Miltenyi Biotech, Bergisch Gladbach, Germany). Cells stimulated with PHA (1 μg/ml, Roche, Switzerland) and with a panel of 176 peptides (15-mers peptides from a broad range of HLA-subtypes and different pathogens (CEFX UltraStim Pool, 1 μg/ml, JPT, Berlin, Germany) provided controls for T-cell functionality and antigen-specific T cells to common viruses. After stimulation, cells were stained with fluorochrome-conjugated monoclonal antibodies in **Table S4** and analyzed by spectral flow cytometry. Responding CD4^+^ and CD8^+^ T cells were identified based on OX40^+^CD137^+^ and CD69^+^CD137^+^ expression, respectively.

***Analysis of Spectral Flow Cytometry Data:*** After acquisition, data preprocessing and QC was done on all samples and time points, including spectral unmixing with autofluorescence subtraction, spill-over correction, and arcsinh transformation (5). Data were then manually gated for removal of atypical events. Samples with viability lower than 35% after 24 hours of *in vitro* stimulation were excluded from the analysis of both flow cytometry panels, although only two of these samples had low (<70%) viability ex vivo. After quality control gating, major immune cell subsets were analyzed by expert gating using FlowJo software version 10.0 (Tree Star Inc., Ashland, OR). Dimensionality reduction and data visualization was performed by combining data for each of 3 groups (healthy controls, and CLL antibody responders and non-responders) using Uniform Manifold Approximation and Projection (UMAP) analysis of the 31-marker panel in OMIQ (Dotmatics, Boston, MA). Cell clusters were identified by FlowSOM analysis. The portions of stimulated T cells with discrete memory phenotypes were analyzed by SPICE (version 6.1) (6). High-dimensional data sets were analyzed using the algorithm T-REX, in order to resolve complex signatures and determine changes in cell populations over time as previously described (<https://github.com/cytolab/T-REX>) (5). For cell regions that significantly expanded or contracted (≤5% and ≥95% change), DBSCAN was used to cluster the cells. Marker Enrichment Modeling from the MEM package (https://github.com/cytolab/mem) was used to assign cellular signatures in regions enriched for discrete molecular features according to quantitative expression of multiple markers, each scored on a scale of 1-10 (7, 8).

***Cytokine Assays***

Culture supernatants from AIM assays were cryopreserved at -80^0^C and analysed by multiplex cytometric bead assay (Human High Sensitivity T Cell Panel (Milliplex, Millipore)) for fractalkine, GM-CSF, IFN-γ, IL1-β, IL-2, IL-4, IL-5, IL-6, IL-7, IL-8, IL-10, IL-12(p70), IL-13, IL-17A, IL-21, IL-23, ITAC, MIP-1α, MIP-1β, MIP-3α, and TNF-α according to the manufacturer’s instructions. Samples were run on a MAGPIX^R^ System (Luminex, Texas). Samples that did not pass quality control were excluded (#61 pre-booster stimulated with PHA and S; #172 and #59 pre-booster stimulated with S). Samples outside limit of detection were assigned the lowest or highest value of the standard curve for each cytokine.

***Statistical Analysis***

Between-group comparisons for normally distributed antibody data were analyzed by Student’s T test and non-normally distributed data were analyzed by Mann-Whitney U test. Frequencies across groups were compared by Fisher’s Exact test. For cellular data, SPICE charts were analyzed by permutation test (6). Comparisons of cellular and cytokine data were performed using a Tukey post-hoc test in a linear mixed model base that accounted for group, response, time point and random effect of subject, and data were adjusted for multiple comparisons.

***Data Sharing Statement***

For original data, please contact gc8jw@virginia.edu or  eca2t@virginia.edu

**References**

1. Keshavarz B, Richards NE, Workman LJ, Patel J, Muehling LM, Canderan G, et al. Trajectory of IgG to SARS-CoV-2 After Vaccination With BNT162b2 or mRNA-1273 in an Employee Cohort and Comparison With Natural Infection. Front Immunol. 2022;13:850987.

2. Ailsworth SM, Keshavarz B, Richards NE, Workman LJ, Murphy DD, Nelson MR, et al. Enhanced SARS-CoV-2 IgG durability following COVID-19 mRNA booster vaccination and comparison of BNT162b2 with mRNA-1273. Ann Allergy Asthma Immunol. 2022.

3. Keshavarz B, Wiencek JR, Workman LJ, Straesser MD, Muehling LM, Canderan G, et al. Quantitative Measurement of IgG to Severe Acute Respiratory Syndrome Coronavirus-2 Proteins Using ImmunoCAP. Int Arch Allergy Immunol. 2021;182(5):417-24.

4. Grifoni A, Weiskopf D, Ramirez SI, Mateus J, Dan JM, Moderbacher CR, et al. Targets of T Cell Responses to SARS-CoV-2 Coronavirus in Humans with COVID-19 Disease and Unexposed Individuals. Cell. 2020;181(7):1489-501 e15.

5. Barone SM, Paul AG, Muehling LM, Lannigan JA, Kwok WW, Turner RB, et al. Unsupervised machine learning reveals key immune cell subsets in COVID-19, rhinovirus infection, and cancer therapy. Elife. 2021;10.

6. Roederer M, Nozzi JL, Nason MC. SPICE: exploration and analysis of post-cytometric complex multivariate datasets. Cytometry A. 2011;79(2):167-74.

7. Diggins KE, Gandelman JS, Roe CE, Irish JM. Generating Quantitative Cell Identity Labels with Marker Enrichment Modeling (MEM). Curr Protoc Cytom. 2018;83:10 21 1-10 21 8.

8. Diggins KE, Greenplate AR, Leelatian N, Wogsland CE, Irish JM. Characterizing cell subsets using marker enrichment modeling. Nat Methods. 2017;14(3):275-8.
