# Supplementary material for: In-depth cellular and humoral dynamics of the response to COVID-19 vaccine booster in patients with chronic B-cell neoplasms
Source: Blood Cancer J. 2023 Jul 26;13(1):114. doi: 10.1038/s41408-023-00884-w (PMC10372059; doi:10.1038/s41408-023-00884-w)
Supplement: Supplementary file 2 — Supplementary Figures and tables [file 41408_2023_884_MOESM2_ESM.pdf]

Supplemental Tables and Figures

In-Depth Cellular and Humoral Dynamics of the Response to COVID-19 Vaccine Booster in Patients with Chronic B-Cell Neoplasms

Emily Ayers, MD [1*], Glenda Canderan, PhD [2*], Michael E Williams, MD [1], Behnam Keshavarz, PhD [2], Craig A Portell [1#], MD, Jeffrey M Wilson ,MD PhD [2#], Judith A Woodfolk, MBChB, PhD [2#]

[1]Division of Hematology and Oncology and Comprehensive Cancer Center, and [2]Division of Asthma, Allergy and Immunology, Department of Medicine, University of Virginia School of Medicine; Charlottesville, VA, USA. Corresponding authors e-mail addresses: eca2t@virginia.edu and gc8jw@virginia.edu

Supplemental Table 1. Characteristics of the NHL Cohort and Vaccine Responder Types.

		Total (n=56)	Responders (>1 µg/mL) (n=27)	Non-responders <1 µg/mL) (n=29)	P value
Age, median (range)		68 (27-88)	68 (27-88)	68 (52-84)	0.29 [a]
Sex, female		21 (38%)	11 (41%)	10 (34%)	0.78 [b]
Disease					
CLL		25 (45%)	15 (56%)	10 (34%)	0.18 [b]
	Treatment naïve	13 (52%)	9	4	
	BTK inhibitor	9 (36%)	3	6	
	Venetoclax based	2 (8%)	2	0	
	No current therapy	1 (14%)	1	0	
DLBCL		5 (9%)	1 (4%)	4 (14%)	0.35 [b]
	Treatment naïve	0	0	0	
	Immunochemotherapy	3 (60%)	1	2	
	No current therapy	1 (20%)	0	1	
	Lenalidomide + rituximab	1(20%)	0	1	
FL		5 (9%)	0 (0%)	5 (17%)	0.05 [b]
	Treatment naïve	0	0	0	
	Anti-CD20 antibody	1 (20%)	0	1	
	Bendamustine based	1 (20%)	0	1	
	No current therapy	3 (60%)	0	3	
MCL		11 (20%)	3 (11%)	8 (38%)	0.18 [b]
	Treatment naïve	1 (9%)	1	0	
	BTK inhibitor	1 (9%)	0	1	
	Anti-CD20 antibody	4 (36%)	0	4	
	No current therapy	1 (9%)	1	0	
	Other	4 (36%)	1	3	
MZL		5 (9%)	5 (19%)	0 (0%)	0.02 [b]
	Treatment naïve	1 (20%)	1	0	
	BTK inhibitor [e]	1 (20%)	1	0	
	Anti-CD20 antibody [e]	2 (40%)	2	0	
	Bendamustine-based	1 (20%)	1	0	
	No current therapy	1 (20%)	1	0	
WM		5 (9%)	3 (11%)	2 (7%)	0.66 [b]
	Treatment naïve	1 (20%)	1	0	
	Anti-CD20 antibody	2 (40%)	1	1	
	No current therapy	2 (40%)	1	1	
Treatment (all disease types)					
	Treatment naïve	16 (29%)	12 (44%)	4 (14%)	0.02 [b]
	No current therapy	9 (16%)	4 (15%)	5 (17%)	>0.99 [b]
	BTKi [d,e]	11 (20%)	4 (15%)	7 (24%)	0.51 [b]
	Anti-CD20 antibody [d]	9 (16%)	3 (11%)	6 (21%)	0.47 [b]
	Other Rx	12 (21%)	5 (19%)	7 (24%)	0.75 [b]
Vaccine type					
	BNT162b2	26 (46%)	12 (44%)	14 (48%)	0.80 [b]
	mRNA-1273	25 (45%)	14 (56%)	11(38%)	0.42 [b]
	Ad26.CoV.S/mRNA-1273	4 (7%)	1 (4%)	3 (10%)	0.29 [b]
	Ad26.CoV.S/ Ad26.CoV.S	1 (2%)	0 (0%)	1 (3%)	>0.99 [b]
Pre-boost sample #		48 (85%)	24 (89%)	24 (83%)	0.71 [b]
	Days pre-boost, median (IQR)	-1.5 (-14 to 0)	-1.5 (-14 to 0)	-1.5 (-14 to 0)	0.76 [c]
	Median S-RBD, µg/mL (IQR)	0.43 (0.09-3.5)	3.5 (1.1-17.4)	0.10 (0.01-0.25)	<0.001 [c]
Early post-boost sample #		56 (100%)	27 (100%)	29 (100%)	>0.99 [b]
	Days early post-boost, median (IQR)	21 (19-26)	21 (19-27)	21 (19-26)	0.78 [c]
	Median S-RBD, µg/mL (IQR)	0.86 (0.14-15.5)	16.6 (5.3-128)	0.16 (0.01-0.36)	<0.001 [c]
Late post-boost sample #		52 (93%)	24 (89%)	28 (97%)	0.34 [c]
	Days late post-boost, median (IQR)	177 (168-189)	183 (172-195)	176 (165-182)	0.03 [c]

[a] Student's T test. [b] Fisher's exact test. [c] Mann-Whitney U test. [d] In Responders: 2 Zanubrutinib, 1 Acalabrutinib, 1 Ibrutinib; Non-responders: 5 Ibrutinib, 2 Acalabrutinib. [e] One MZL patient was treated with Zanubrutinib and CD20 concurrently.

Antibody responses to vaccine were assessed in all subjects. "No current therapy" denotes subjects who received no treatment within 6 months from the booster vaccination.

Supplemental Table 2. Comparison of NHL and Employee Reference Cohort.

	NHL cohort (n=56)	Employee reference cohort (n=28)	P-value
Age, median (range)	68 (27-88)	63 (51-87)	0.19 [a]
Sex, female	21 (38%)	17 (61%)	0.07 [b]
BNT162b2	26 (46%)	13 (46%)	>0.99 [b]
mRNA-1273	25 (45%)	14 (50%)	0.65 [b]
Other	5 (9%)	1 (4%)	0.66 [b]
Pre-boost sample #	48 (85%)	17 (61%)	0.02 [b]
Days pre-boost, median (IQR)	-1.5 (-14 to 0)	-2.0 (-5.5 to -0.5)	0.91 [c]
Median S-RBD pre-boost, µg/mL (IQR)	0.43 (0.09-3.5)	3.3 (1.7-4.6)	0.005 [c]
Early post-boost sample #	56 (100%)	28 (100%)	>0.99 [b]
Days early post-boost, median (IQR)	21 (19-26)	21 (19-30)	0.87 [c]
Median S-RBD early post-boost, µg/mL (IQR)	0.86 (0.14-15.5)	62.2 (30-99)	<0.001 [c]
Late post-boost sample #	52 (93%)	25 (89%)	0.68 [c]
Days late post-boost, median (IQR)	177 (168-189)	133 (127-146)	<0.001 [c]
Median S-RBD late post-boost, µg/mL (IQR)	2.5 (0.10-31.2)	23.5 (12-71)	<0.001 [c]

[a] Student's T test. [b] Fisher's exact test. [c] Mann-Whitney U test.

Supplemental Table 3. Characteristics of Patients Included in Cellular Studies.

GROUPS	Ab Responder/ Non-Responder[a]	ID	Vaccine regimen	Days pre-booster	Days post-booster	Pre-booster Spike Ab (µg/ml)	Post-booster Spike Ab (µg/ml)	Fold Change Spike Abs	Age	Sex	Stage[b] (Time from diagnosis)	Treatment (Duration of treatment)
CLL	NR	17	Moderna	-62	22	0.05	0.08	2	71.3	F	IV (9 yrs)	BTKi Ibrutinib (2 yrs)
	NR	33	Pfizer	-8	12	0.27	0.31	1	74	F	IV (15 yrs)	BTKi Ibrutinib (8 yrs)
	NR	55	Moderna	-23	27	0.27	0	0	65.5	M	III (8 yrs)	BTKi Acalabrutinib (3 yrs)
	NR	59	Pfizer	-17	20	0.48	0.83	2	52	M	II (4 yrs)	None
	NR	64	Pfizer	-17	19	0.48	0.88	2	63	M	IV (3 yrs)	None
	NR	50	Pfizer	-4	40	0.82	2.54	3	63.1	F	I (8 yrs)	None
	R	60	Pfizer	-16	21	1.5	95.01	63	62.1	F	I (1 yrs)	None
	R	52	Pfizer	-1	19	3.02	211.53	70	78.1	M	0 (6 yrs)	None
	R	61	Moderna	-17	25	4.44	17.93	4	72.4	F	I (3 yrs)	None
	R	41	Pfizer	0	21	5.22	50.08	10	78.3	M	0 (7 yrs)	None
	R	53	Pfizer	-3	26	8.92	198.34	22	88.3	F	I (23 yrs)	None
	R	30	Moderna	0	15	39.55	313.97	8	71.8	M	I (1 yrs)	None
HEALTHY	R	28	Pfizer	-1	20	3.26	106.4	33	64	F	NA	None
	R	159	Moderna	-49	21	4.9	18.16	4	76	M	NA	None
	R	172	Moderna	-2	19	15.07	62.27	4	53	M	NA	None

[a] Non-responders were defined as patients with IgG to S-RBD below 1 µg/ml before vaccine booster who had limited post-booster responses (IgG antibodies < 3 µg/ml with < 4-fold change).

[b] Disease stage at time of diagnosis.

Data shown for 12 patients with CLL and 3 healthy controls. IgG antibody levels are those present at the time that cellular studies were performed.

Supplemental Table 4. Marker Panels for Spectral Flow Cytometry.

A.

MARKER	FLUOROPHORE	CLONE	Cat #	Company
CCR7	BV480	3d12	566099	BD
Live dead	Blue	NA	L23105	Thermo Fisher
CD21	BUV395	b-ly4	740288	BD
CD15	BUV496	w6d3	741187	BD
CD27	BUv661	o323	751680	BD
HLA-DR	BUV805	g46-6	748338	BD
TCRα24	BV421	6b11	342916	BioLegend
CD123	SB436	6H6	62-1239-42	Thermo Fisher
CD16	Pacific blue	3G8	mhcd1628	Thermo Fisher
CD14	BV510	M5E2	301842	BioLegend
CD8	BV570	RPA-T8	301038	BioLegend
CD1c	BV605	L161	331538	BioLegend
CD56	BV650	HCD56	318344	BioLegend
CD19	BV711	SJ25C1	363022	BioLegend
CD4	BV750	SK3	344644	BioLegend
CD28	BV785	CD28.2	302950	BioLegend
CD11c	BB515	bu-15	566835	BD
CD45RA	Alexa 488	HI100	304114	BioLegend
CD3	Alexa 532	UCHT1	58-0038-42	Thermo Fisher
CD33	Percp	wm53	a15804	Thermo Fisher
CD11b	PerCP-Cy5.5	ICRF44	301328	BioLegend
TCRγδ	BB700	11f2	745944	BD
CD117	PE	104d2	313204	BioLegend
IgD	PE-Dazzle	IA6-2	348240	BioLegend
CD95	PE-Cy5	DX2	305610	BioLegend
CD25	PEAF700	3G10	mhcd2524	Thermo Fisher
CRTH2	PE-Cy7	bm16	350118	BioLegend
TCR Vβ11	APC	c21	a66905	beckman
CD57	eFluor 660	TB01	50-0577-42	Thermo Fisher
CD127	APC-R700	HIL-7R-M21	565185	BD
CD38	APCfire810	hit2	303550	BioLegend

B.

MARKER	FLUOROPHORE	CLONE	Cat #	Company
Live/dead	Blue	NA	L23105	Thermo Fisher
CD3	SB550	SK7	344852	BIOLEGEND
CD4	BUV496	OKT4	750980	BD
CD8	BUV805	RPA-T8	749366	BD
CD45RA	BV605	HI100	304133	BIOLEGEND
CCR7	APC fire750	G043H7	353246	BIOLEGEND
CD27	BV570	323	302825	BIOLEGEND
CCR5	BUV737	2D7	565293	BD
OX40	PECY7	Ber-ACT35	350012	BIOLEGEND
CD137	APC	4B4-1	309810	BIOLEGEND
CD69	PECF594	FN50	562617	BD

(A) 31-marker panel for high-dimensional single-cell analysis of immune cells in the blood.
(B) Marker panel used to identify Ag-specific T cells by activation induced marker (AIM) assay.

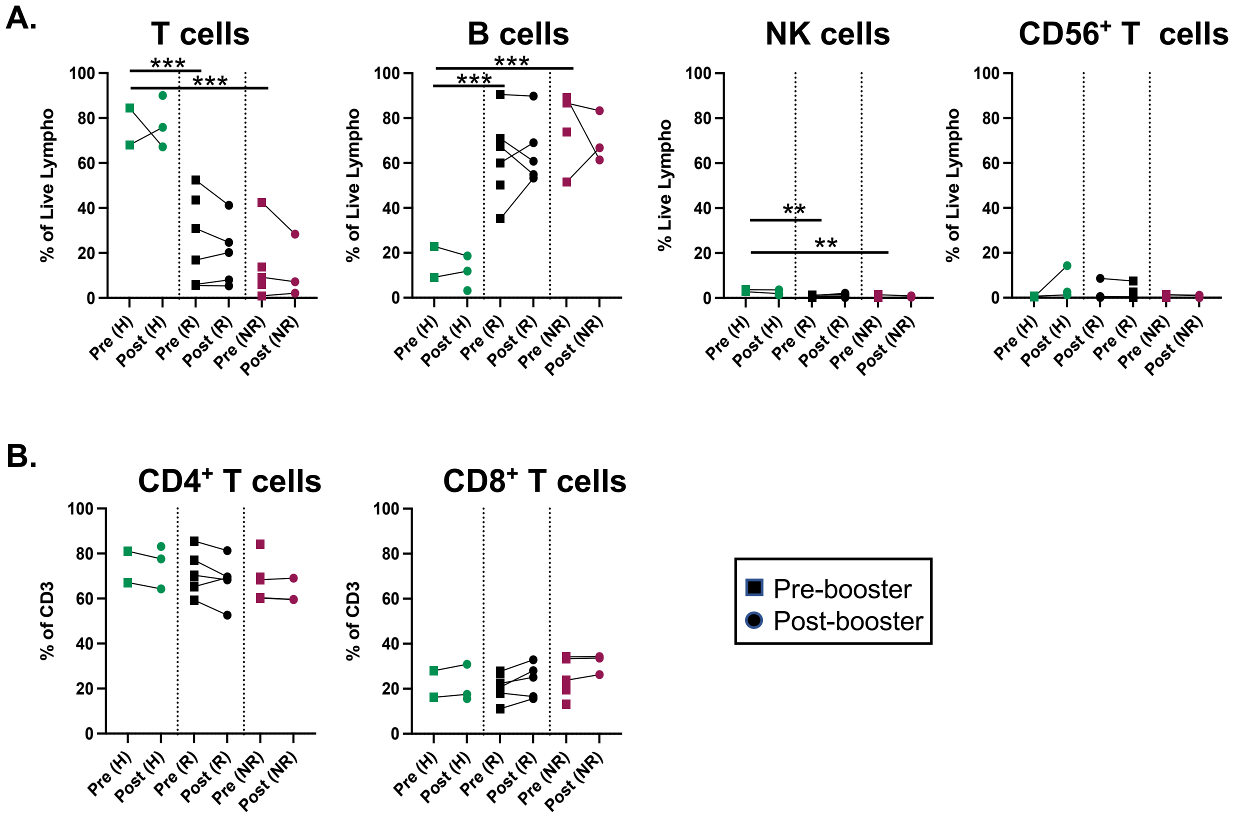

Supplemental Figure 1. Percentages of Major Lymphocyte Subsets in the Blood by Manual Gating. For each subject, cell subsets were analyzed at available time points pre- and post-booster. **(A)** Percentages of total T cells (CD3+), B cells (CD19+), natural killer (CD56+) cells and CD56+ T cells within the lymphocyte compartment. **(B)** Percentages of CD4+ and CD8+ T cells within the T-cell compartment. Data is shown for healthy subjects (H, *green*), CLL responders (R, *black*), and CLL non-responders (NR, *magenta*). Data from subjects #50 and #159 (pre-booster), and #50, #59, #60 and #64 (post-booster) were excluded from the analysis owing to low viability. Data was analyzed by Tukey post-hoc test in a linear mixed model. ** $p<0.01$, ***$p<0.001$.

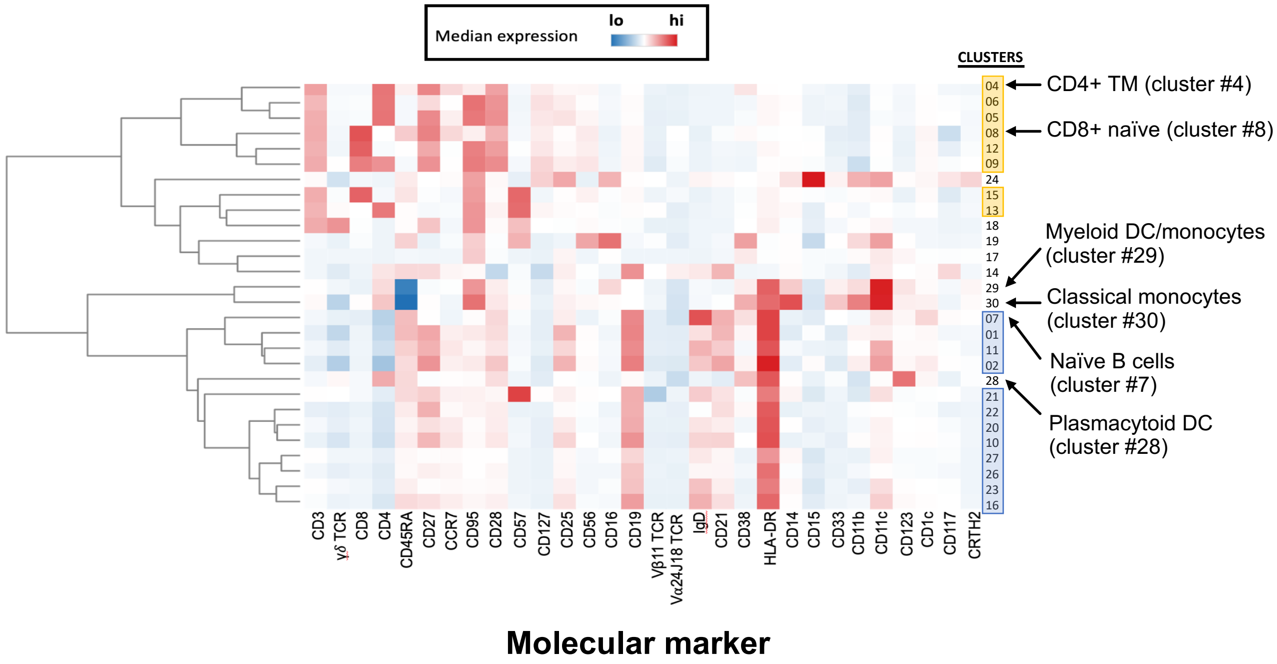

Supplemental Figure 2. Heatmap of the median expression of 30 markers across the 28 clusters identified by FlowSOM. Each column of the heatmap denotes the median expression of the markers included in the spectral flow cytometry panel, and each row corresponds to each of the 28 cell clusters. Clusters whose frequencies were significantly different between CLL and healthy groups are denoted by an arrow. T and B cell populations are denoted by orange and blue boxes respectively.

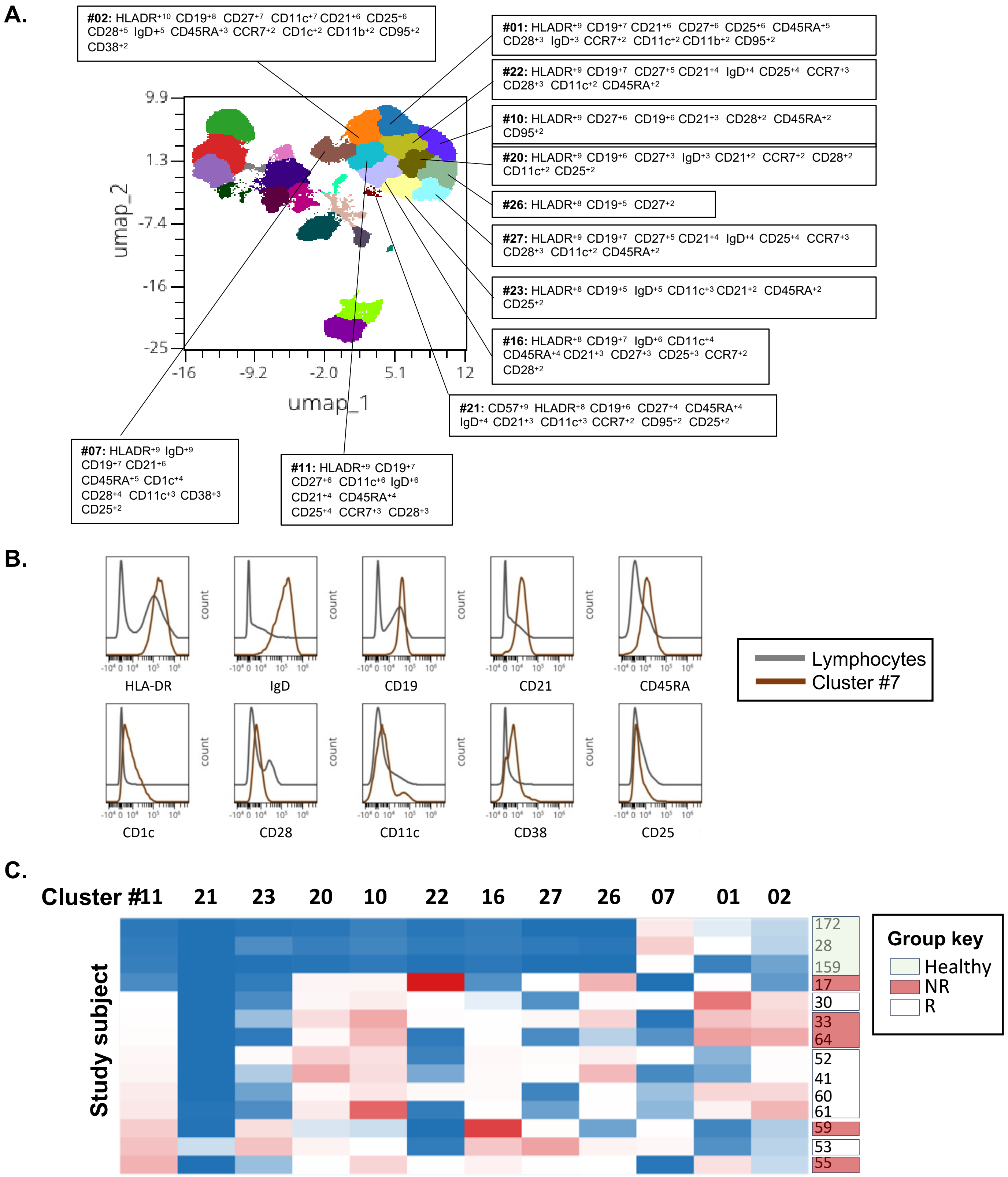

Supplemental Figure 3. Signatures of B-cell Clusters Identified by Marker Enrichment Modeling. (A) FlowSOM analysis identified 28 cell clusters according to their discrete molecular signatures generated using a 31-marker panel for spectral flow cytometry. Signatures of cell clusters were assigned by marker enrichment modeling (MEM), which scores each marker on a scale of 1-10 based on its enrichment within each cluster. **(B)** Histograms of fluorescence intensity for each MEM marker expressed on B-cell cluster #7 overlaid on histograms for the same marker expressed on total lymphocytes. **(C)** Heatmap of the abundance of each B-cell cluster by subject. Each row represents a single subject and each column represents a B-cell cluster. Numbers in right column denote subject ID#. Cell samples obtained before and after vaccine booster were concatenated by time point when both were available.

A.

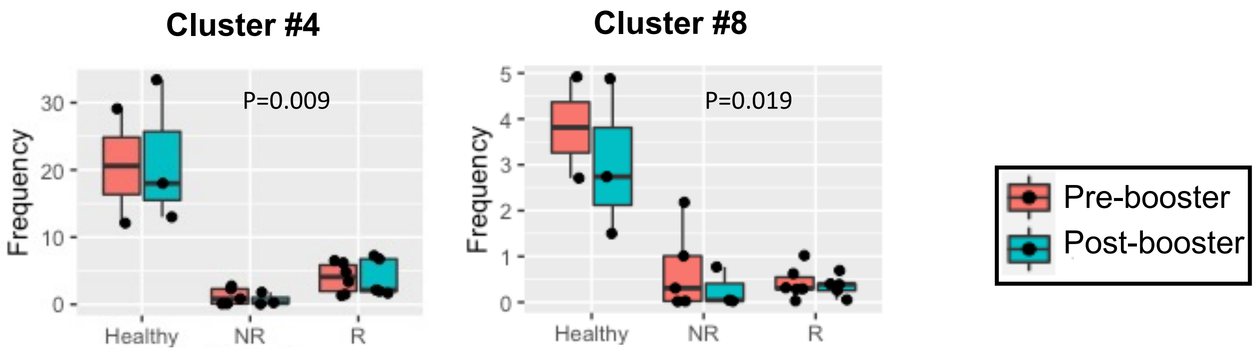

B.

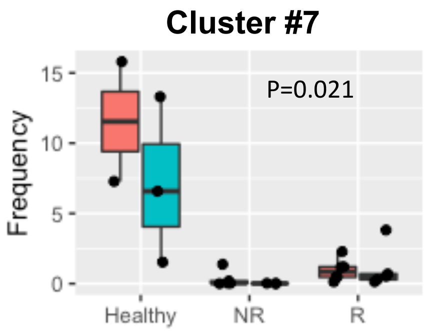

CLUSTER #	CELL SUBSET	CLUSTER MEM SIGNATURE											
4	TM CD4 T	$CD4^{+8}$	$CD27^{+7}$	$CD28^{+6}$	$CD3^{+6}$	$CCR7^{+4}$	$CD38^{+3}$	$CD45RA^{+2}$	$CD95^{+2}$	$CD127^{+2}$			
7	Naïve B	$HLADR^{+9}$	IgD^{+9}	$CD19^{+7}$	$CD21^{+6}$	$CD45RA^{+5}$	$CD1c^{+4}$	$CD28^{+4}$	$CD11c^{+3}$	$CD38^{+3}$	$CD25^{+2}$		
8	Naïve CD8 T	$CD8^{+9}$	$CD27^{+8}$	$CD28^{+6}$	$CD3^{+6}$	$CCR7^{+4}$	$CD45RA^{+4}$	$CD95^{+2}$	$CD127^{+2}$				
28	pDC	$HLADR^{+9}$	$CD123^{+8}$	$CD4^{+6}$	$CD38^{+5}$	$CD45RA^{+4}$							
29	Myeloid DC/monocytes	$CD11c^{+10}$	$HLADR^{+8}$	$CD95^{+7}$	$CD16^{+4}$	$CD14^{+4}$	$CD123^{+3}$	$CD4^{+3}$	$CD11b^{+3}$	$CD33^{+2}$	$CD38^{+2}$		
30	Classical Monocytes	$CD11c^{+10}$	$CD14^{+9}$	$HLADR^{+8}$	$CD11b^{+8}$	$CD95^{+8}$	$CD38^{+6}$	$CD4^{+5}$	$CD33^{+5}$	$CD45RA^{+4}$	$CD123^{+2}$	$CD1c^{+2}$	$CD25^{+2}$

C.

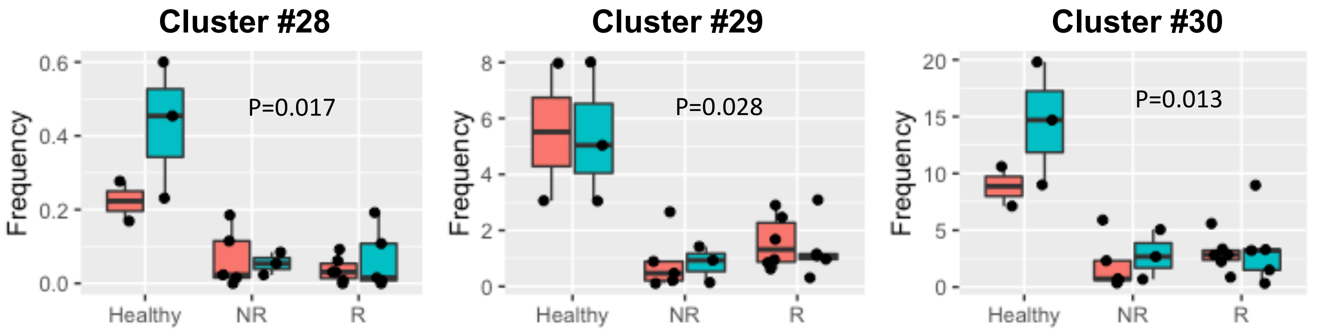

Supplemental Figure 4. Percentages of Cell Clusters that Differed Between CLL Patients and Healthy Subjects. Clusters included T-cell subsets **(A)**, B-cell subsets **(B),** and innate cell types **(C)**. Data are shown for 3 healthy subjects, and 6 non-responder and 6 responder CLL patients. Data for subjects #50 and #159 (pre-booster), and #50, #59, #60 and #64 (post-booster) were excluded from the analysis owing to low viability. The statistical test used was Tukey's test for post hoc analysis in a linear mixed model. Signatures assigned by MEM labeling for each cell cluster are shown in the text box.

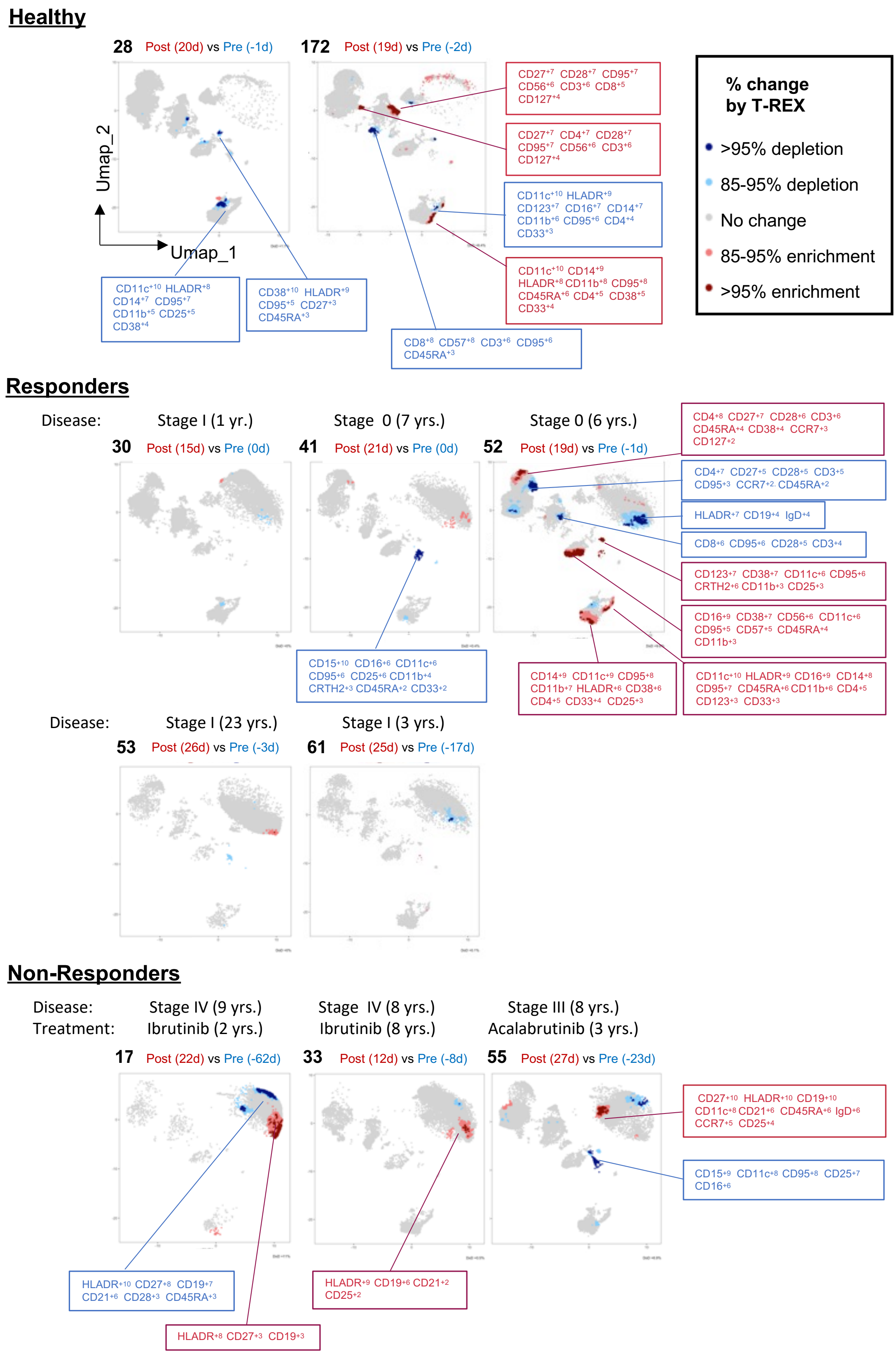

Supplemental Figure 5. Changes in Cell Populations in the Blood After Vaccine Booster in Responder and Non-Responder CLL Patients. T-REX analysis is shown for 5 responder CLL patients, 3 non-responder CLL patients, and 2 healthy controls. Values in parentheses related to disease and treatment are for duration of disease and time on current treatment respectively. Enrichment (red shading) and depletion (blue shading) of discrete cell clusters is depicted according to the percentage change for each cluster. MEM labeling was used to assign signatures to populations of interest identified by T-REX, according to quantitative expression of multiple markers, each scored on a scale of 1-10. Analyses were performed in subjects with high viability specimens available at pre- and post-booster time points.

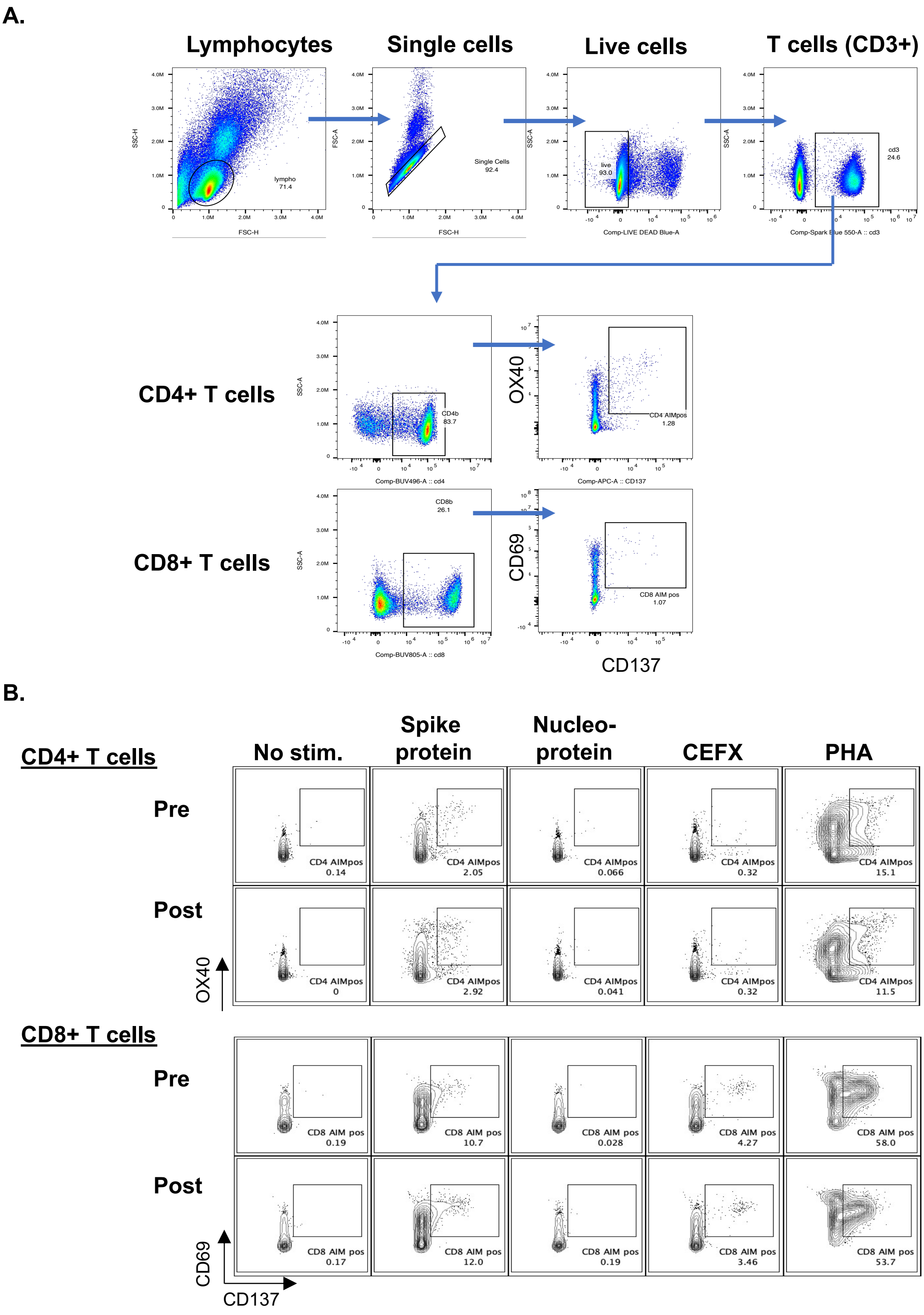

Supplemental Figure 6. Gating Strategy to Identify Virus-Specific CD4+ and CD8+ T Cells by Activation-Induced Marker (AIM) assay. T cells responding to SARS-CoV-2 proteins were identified after a 24 hours of *in vitro* stimulation with 15-mer peptides spanning the entire length of spike glycoprotein and nucleoprotein. Conditions stimulated with PHA and 15-mers from common pathogens (CEFX) were included as positive controls for T-cell function. **(A)** Gating strategy to identify antigen-specific T cells. Responding $CD4^{+}$ and $CD8^{+}$ T cells were identified based on $OX40^{+}CD137^{+}$ and $CD69^{+}CD137^{+}$ expression respectively. **(B)** Representative data from one responder CLL (subject #30) subject showing results for each stimulation condition before and after vaccine booster. The presence of signals for spike glycoprotein but not nucleoprotein is consistent with vaccine-induced T-cell responses to SARS-CoV-2.

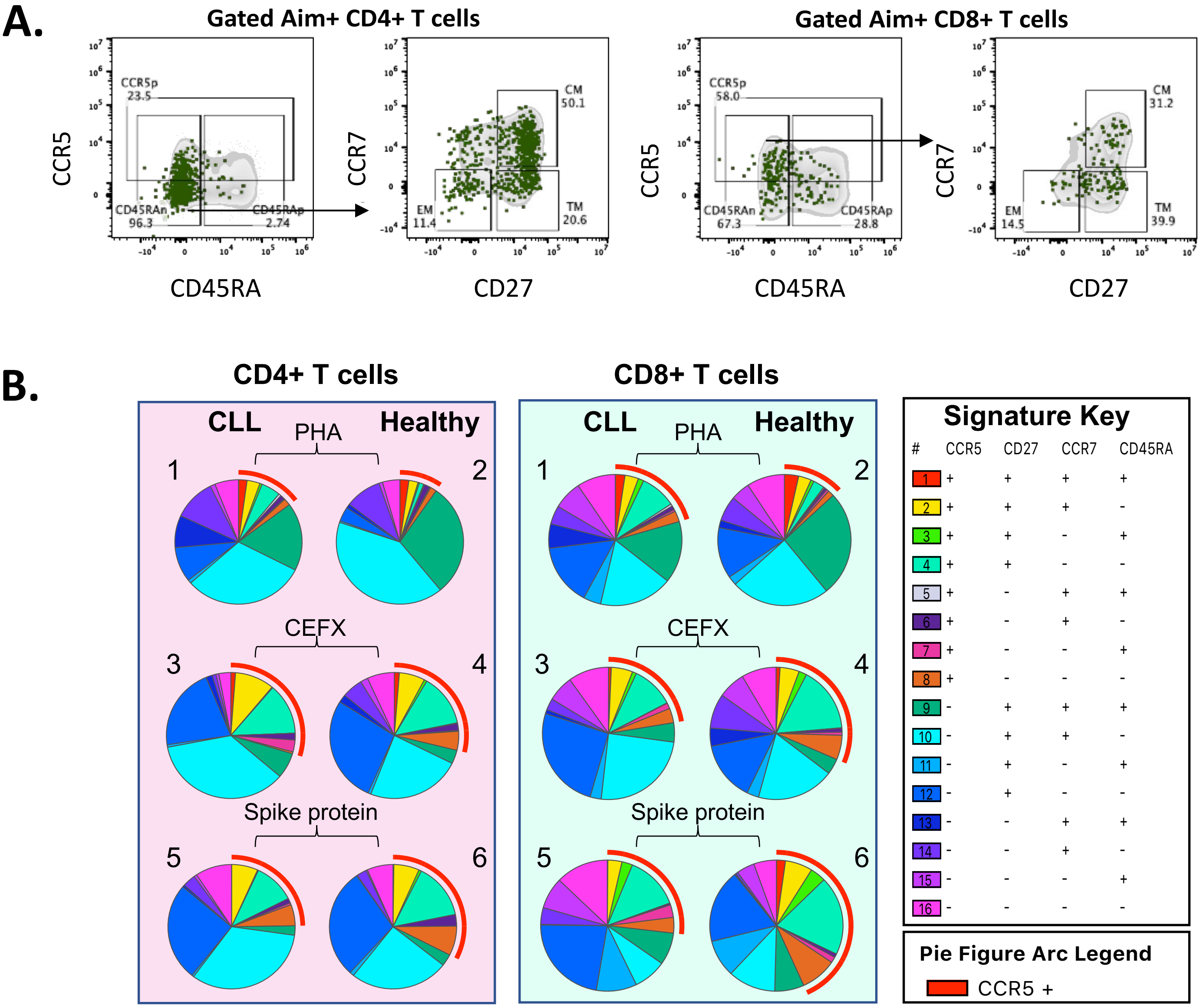

p-values for comparisons of SPICE charts.

#	1	2	3	4	5	6
1		0.0014	0.0031	<0.0001	0.0001	<0.0001
2	0.0014		0.0032	0.0038	<0.0001	0.0023
3	0.0031	0.0032		0.5748	0.5873	0.5020
4	<0.0001	0.0038	0.5748		0.6721	0.8805
5	0.0001	<0.0001	0.5873	0.6721		0.6721
6	<0.0001	0.0023	0.5020	0.8805	0.6721	

#	1	2	3	4	5	6
1		0.0415	0.0576	0.0920	0.0885	0.0032
2	0.0415		0.0164	0.0181	0.0066	0.0092
3	0.0576	0.0164		0.5238	0.1650	0.1619
4	0.0920	0.0181	0.5238		0.7278	0.4064
5	0.0885	0.0066	0.1650	0.7278		0.9616
6	0.0032	0.0092	0.1619	0.4064	0.9616	

Supplemental Figure 7. Surface Signatures of Virus-Specific T Cells. **(A)** Representative scatter plots depicting expression of memory markers (CD45RA, CD27) and chemokine receptors (CCR5 and CCR7) on spike protein-specific CD4+ and CD8+ T cells after vaccine booster (subject #55). **(B)** SPICE charts showing the proportions of distinct signatures of CD4+ and CD8+ T cells present in AIM assays after 24 hours of antigen stimulation based on expression of CD45RA, CD27, CCR5, CD27. Each slice of the "pie" denotes a different signature colorized according to the key in the panel on the right side. Spice charts show data compiled for all samples from CLL patients (left, n=19) and healthy subjects (right, n=5). Pie charts are shown for each of 3 stimulation conditions: PHA, CEFX peptide pool and spike protein peptide pool. Data for samples #50 and #159 (pre-booster), #50, #59, #60, and #64 (post-booster) were excluded from the analysis owing to low viability. Differences in SPICE charts were analyzed by permutation test and the resulting p-values for each chart comparison (numbered 1-6) are shown in tables underneath the spice charts. Significant values are highlighted in red (p<0.05).

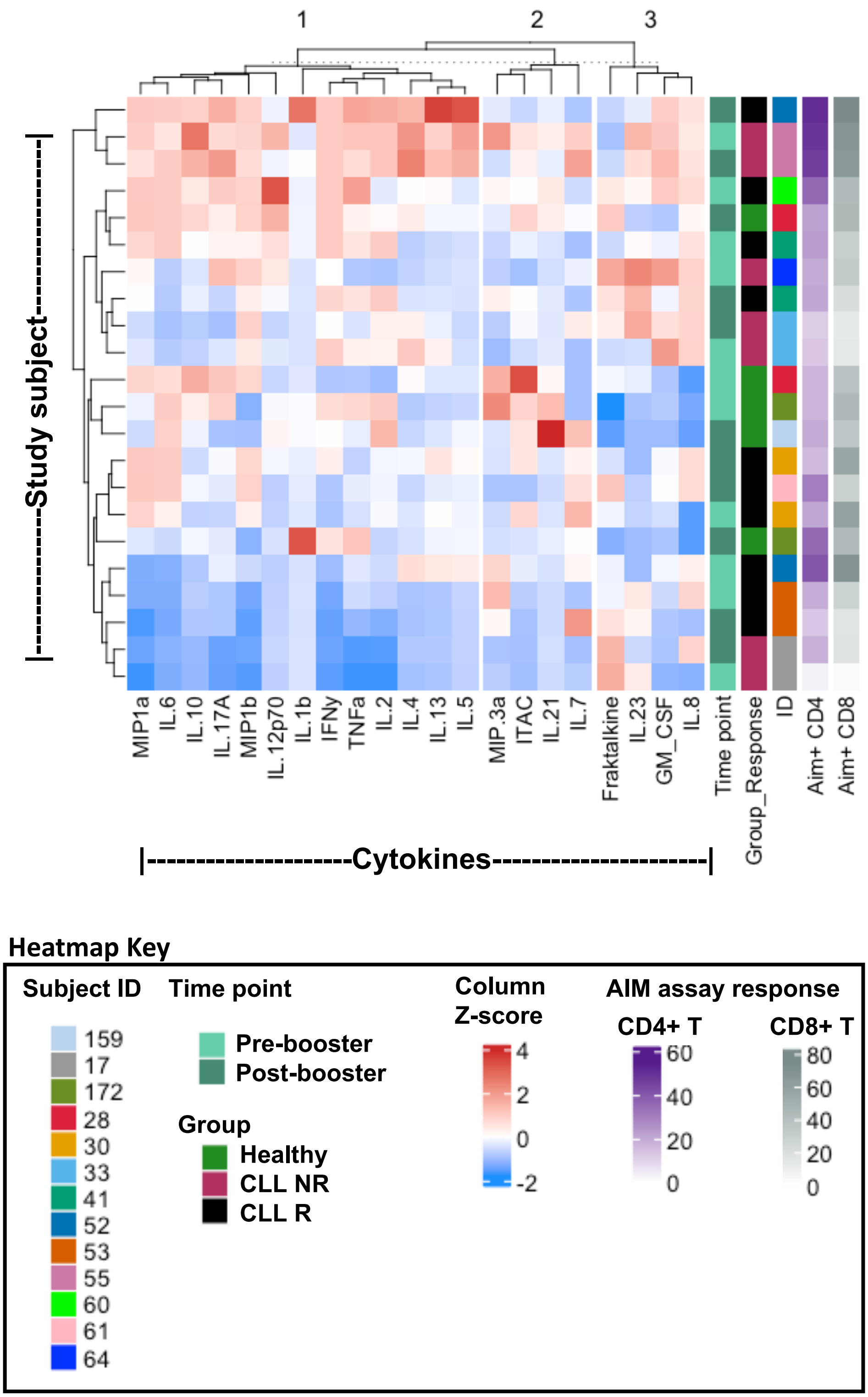

Supplemental Figure 8. Cytokine Response to PHA in AIM Assays. Heatmap showing z-scaled cytokine levels (pg/ml) in AIM assay supernatants harvested after stimulation with PHA. Values for unstimulated conditions were subtracted before scaling. Data for samples #50 and #159 (pre-booster), #50, #59, #60, and #64 (post-booster) were excluded owing to low cell viability. Data for 3 pre-booster samples (#59 and #61) were excluded from analysis of cytokine data for technical reasons.
